# Subjective Wellbeing in Rural China: How Social Environments Influence the Diurnal Rhythms of Affect

**DOI:** 10.3390/ijerph18084132

**Published:** 2021-04-14

**Authors:** Jiyao Sun, Nan Zhang, Bram Vanhoutte, Jian Wang, Tarani Chandola

**Affiliations:** 1 Social Statistics, Manchester Institute for Collaborative Research on Ageing (MICRA), The University of Manchester, HBS Building, Oxford Road, Manchester M13 9PL, UK; jiyao.sun@manchester.ac.uk (J.S.); nan.zhang-2@manchester.ac.uk (N.Z.); bram.vanhoutte@ulb.ac.be (B.V.); tarani.chandola@manchester.ac.uk (T.C.); 2 Cathie Marsh Institute for Social Research (CMI), The University of Manchester, HBS Building, Oxford Road, Manchester M13 9PL, UK; 3 Center for Health Management and Policy Research, School of Public Health, Cheeloo College of Medicine, Shandong University, Jinan 250012, China; 4 NHC Key Lab of Health Economics and Policy Research, Shandong University, Jinan 250012, China; 5 École de Santé Publique, Université Libre de Bruxelles, Route de Lennik 808-CP591, 1070 Brussels, Belgium

**Keywords:** diurnal rhythm, affect, subjective wellbeing, rural China, day reconstruction method

## Abstract

Although the diurnal rhythms of affect influence people’s health and behavior, there is a lack of evidence from rural China, where the types and timing of social activities may differ from Western contexts. In this study, a total of 2847 Chinese rural residents from three provinces of China are interviewed using the abbreviated Day Reconstruction Method (DRM) questionnaire. Diurnal rhythms of three affective subjective wellbeing (SWB) indicators—positive affect (PoA), negative affect (NeA), and net affect are analyzed by multilevel models. Our results show PoA and net affect generally increase in magnitude throughout the day with two peaks around noon and in the evening, respectively; whereas, there is an overall decline in NeA as the day passes with two troughs occurring at lunchtime and in the evening. These patterns, however, flatten considerably, with the lunchtime peaks in PoA and net affect (and trough in NeA) disappearing entirely, after further controlling for two social environmental factors—activity type and the quality of social interaction. This study, set in rural China, corroborates the diurnal rhythms of affect from prior Western research to some extent, and highlights that social environmental factors have a significant effect on diurnal rhythms of affect in the rural Chinese context. It is possible that the diurnal rhythms of affect could change in response to stimulation from the environment. Improving some social environmental factors, such as organizing pleasant activities and creating a friendly interactive environment, could contribute to the increase in positive affect and decline in negative affect, thereby enhancing the quality of life.

## 1. Introduction

Although there are many indicators that reflect the quality of life, one key perspective is one’s own subjective evaluation—a construct known as subjective wellbeing (SWB) [1]. Diener [2] defined SWB as consisting of overall life evaluations, as well as day-to-day emotions. As an expansive and multidimensional term, SWB comprises both cognitive (e.g., life satisfaction) and affective components (e.g., happiness, sadness) [3,4]. Scholars emphasize that the affective component of SWB, which consists of moods and emotions, both positive and negative [5], can reflect fundamental qualities of experience and plays a pivotal role in understanding and predicting human health and behavior [6]. Some researchers measure affective SWB retrospectively, by asking a single question, like “Taking all things together, how would you say that you are these days: very happy, pretty happy, or not too happy?” [7], or using rating scales, such as the Positive (e.g., cheerful) and Negative (e.g., irritable) Affect Schedule, where people rate the intensity of several emotions over a specific time frame [8]. Others prefer to measure affective SWB by capturing actual momentary affective states experienced in daily life [9], because global retrospective measures could be biased by personality traits, current mood, and limits on the number of recalled memories [10]. Regardless of the affective SWB measurements, most studies have focused on between-person differences in the levels of affect, either in general or at particular points in time, often overlooking the importance of the rhythm of affect over the course of the day [6].

Research on these diurnal rhythms of affective SWB is worthwhile and meaningful, as they play a vital predictive role in people’s health and behavior. First, specific diurnal rhythms of affect have been linked to depression. For example, Peeters et al. [11] examined the diurnal variation of positive affect (PoA) and negative affect (NeA) among individuals with and without major depressive disorder (MDD), and reported a distinct diurnal rhythm of affect in the MDD group. Relative to healthy subjects, PoA in participants with MDD was characterized by lower overall levels, reduced interindividual variation, and a delayed peak, while there were higher overall levels of NeA with both greater interindividual and moment-to-moment variance. Specific diurnal patterns of affect could also be found in patients with other psychiatric disorders [12], which shows clinical potential for tracking the severity of mental disorders [11]. Second, the diurnal patterns of affect are likely to predict physical diseases. There is considerable evidence that increased negative affect, such as depression and stress, is associated with an increased risk of premature mortality, coronary heart disease, disability, and other chronic disorders [13], while frequent high levels of positive affect have been found to protect against poor health [14], and predict lower future mortality and morbidity rates [15]. Additionally, diurnal patterns of affect have been shown to be a strong predictor of suicidal ideation. Tian and colleagues [16] explored the diurnal variation of positive feelings between Chinese working women with and without suicidal ideation. They found that women with suicidal ideation demonstrated a significantly lower level of and higher volatility in positive feelings, and suggested that more attention should be paid to the role of diurnal rhythms of positive moods in female suicide prevention research and intervention design in the future. Therefore, research on the diurnal rhythms of affect could be helpful in understanding and preventing poor health, and improving quality of life.

Diurnal rhythms of affect are likely to be a product of both social environmental factors and physiological processes [6]. In relation to social environmental factors, it is likely that different types of activities impact affect differently depending on the time of day. For example, regular daily activities such as working and watching TV are likely to be associated with increased negative and positive feelings, respectively [17]. Research by Stone et al. [6] reported bimodal diurnal patterns for both positive and negative emotions in 909 American working women, with positive affect reaching peaks at noon and evenings, while peaks in negative affect were observed at mid-morning and mid-afternoon. They interpreted the bimodality of diurnal patterns in the function of different activity types: The increase in negative emotions was attributed to work, while lunch break provided a pleasant environment and a respite from the demands of the work, reducing negative emotions and increasing positive emotions. When the study considered the effects of activity types, diurnal patterns of affect flattened considerably with the lunchtime peak in positive affect and trough in negative affect entirely eliminated, indicating the important contribution of social activities to diurnal patterns. Another important social environmental factor that might influence people’s diurnal rhythms of affect could be the quality of social interaction when engaging in activities. Research by Lee et al. [18] among participants in the World Health Organization (WHO) Study on Global Ageing and Adult Health (SAGE) examined the association of the quality of social interaction and time of day (ToD) on net affect scores. They found that compared with activities that participants experienced alone, activities that were accompanied by friendly interacting partners were associated with higher net affect scores, while having irritating interacting partners produced lower net affect scores. Moreover, diurnal rhythms of affect may also be influenced by diurnal endogenous factors (e.g., cortisol, growth hormone) [19]. For instance, cortisol declines steeply throughout the day from a morning peak [20]. Positive affect has been shown to be associated with reduced cortisol output during the day [21,22]. Therefore, the diurnal rhythm of affect might be particularly influenced by some psychosocial environmental factors, or more susceptible to physiological processes [23,24].

From a methodological perspective, several instruments are available to measure actual momentary affective states, and construct diurnal patterns of affect, such as the Experience Sampling Method (ESM) [25], Ecological Momentary Assessment (EMA) [26], and the Day Reconstruction Method (DRM) [17]. Although ESM/EMA are regarded as the gold standard for studying diurnal patterns of momentary mood and experience in daily life, reducing heuristic and recall biases while maximizing ecological validity [27], it has inevitable practical limitations. For example, an ESM/EMA study relying on electronic equipment can be costly [17]. On the other hand, an ESM/EMA study asks participants to report their emotions several times a day, which can be burdensome and intrusive [28]. It is sometimes impossible for respondents to answer the survey at the moment of contact (e.g., during an intense argument, or for high-stress occupational groups), leading to measurement bias [1,29]. DRM (Kahneman et al. [17]) can overcome the drawbacks of ESM/EMA and construct very close diurnal patterns of affect to those generated by ESM/EMA [17,29,30]. Researchers have argued that the original version of the DRM questionnaire may be challenging during large-scale administration, because it is time-consuming and usually takes from 45–75 min to complete [17]. An abbreviated version of the DRM questionnaire has been created, which only takes 15–25 min to complete, as time is at a premium during large-sample investigations. This abbreviated version of the DRM has been validated in the SAGE study [31], and the Collaborative Research on Ageing in Europe (COURAGE) study [32]. The abbreviated DRM has shown to have adequate reliability [31,32,33,34], construct validity [33,34], temporal stability (test-retest) [35], and measurement invariance [32] in previous studies.

Nearly all the studies on the diurnal rhythms of affect have been in Western contexts, such as in the US [6,17], Germany [1], and the UK [29], and in working populations [6,17,29]. Based on these studies, there is a general consensus that positive affect increases throughout the day, whereas negative affect tends to decrease for most of the day. Moreover, Stone et al. [6] observed bimodal diurnal rhythms for both positive and negative emotions. However, there is a lack of evidence from non-Western contexts with different cultural attitudes and social contexts. A survey of the top psychology journals found that 96% of subjects in scientific studies were from Western industrialized countries—which include just 12% of the world’s population [36]. The diurnal rhythms of affect depicted in Western, educated, industrialized, rich, and democratic (WEIRD) societies may differ very little from each other in terms of both living conditions and cultural backgrounds. This presents a challenge for comparisons among differing societies [37]. Nevertheless, in less developed countries, with fewer resources and capacities at their disposal, diurnal rhythms of affect are understudied. Further research on diurnal patterns of affect in non-western, low- and middle-income countries, such as China, the largest developing country in the world, would provide useful comparisons to Western societies [33].

Indeed, the past decade has witnessed an emerging volume of literature exploring SWB in China [38]. Research has focused on urban areas [39,40], the elderly [41,42,43], and generally investigates the relationship between SWB and socio-economic status [40,44,45,46] or health [38,47]. Although some scholars, such as Knight et al. [48], have studied the SWB of rural Chinese, to our knowledge, research on fine-grained diurnal rhythms of affective SWB among rural Chinese has not yet been conducted. Devoting effort to studying diurnal rhythms of affective SWB of rural residents in China is worthwhile for several reasons. First, when the data collection of the present study was carried out in 2006, the National Bureau of Statistics of China (NBS) showed that more than half of the Chinese population (55.66%) were living in rural areas [49]. The latest data from 2019 illustrate that China is still more than one-third rural (39.40%) [50]. Although rural Chinese still make up a significant portion of the population, the current SWB literature provides limited data regarding this subgroup [38]. Second, it is acknowledged that the Chinese economy is characterized by a remarkable rural-urban divide, largely owing to policies favoring the urban context. For example, the policy of urban-rural household registration system, in which every Chinese citizen has been assigned the agricultural (rural) or non-agricultural (urban) hukou at birth according to the residential location since 1958, has served as an overriding basis to allocate resources in favor of city-dwellers to facilitate industrialization and maintain urban stability, which contributes to a great rural-urban disparity in social economic status and in the entitlement to social welfare [48], and has shown to have long term consequences in terms of mental wellbeing [51]. Compared with their urban counterparts, Chinese rural dwellers are living in greater socioeconomic disadvantage [52]. Given the evidence that diurnal rhythms of affect have a significant impact on health and wellbeing, conducting research among rural residents can provide some ground to shift policy priorities to improve rural living standards and health. Third, due to the distinct sociocultural contexts and lifestyles, exploring if the diurnal rhythms of affect observed in Western population can be translated into the rural Chinese context is needed. However, no existing literature, to our knowledge, has examined this topic in a rural Chinese population.

This stud aims to examine the diurnal rhythms of affective SWB as measured by PoA, NeA, and net affect. First, we describe the diurnal rhythms of affect after controlling for the county of residence, sets of DRM questionnaire, and sociodemographic factors in statistical models. We then further control for two social environmental factors—activity type and the quality of social interaction—in the models to examine whether the diurnal patterns of affect remain when the influence of social environmental factors is taken into account.

## 2. Data and Methods

### 2.1. Data

The data used came from the cooperative project “The evaluation of subjective well-being based on DRM in rural China” between Shandong University, China, and Harvard University, USA. Several studies have already been successfully published in peer-reviewed journals using this dataset [46,47,53,54].

The data collection was conducted in July and August 2006, and timed to avoid intensive farming times, such as harvest or planting. Three provinces of China were selected: Shandong, An’hui, and Sichuan, according to levels of socioeconomic development and geographic location. These provinces (in the above order) are located in the east coastal, central, and southwestern regions of China with a per capita gross domestic product (GDP) of 20,443 yuan, 10,630 yuan, and 10,371 yuan based on 2006 statistics [55]. Four counties were chosen from these provinces: Caoxian and Chiping (Shandong), Linquan (An’hui), and An’yue (Sichuan). Within each county, a stratified, multistage cluster random sampling design was used to select townships and villages. First, four townships were chosen based on levels of socioeconomic development and geographic location from each county; second, four villages were selected in a similar manner from each township. Subsequently, systematic sampling was employed to choose households based on the hukou (household) registration in the villages, and 25–30 households were visited within each village. Rural residents aged between 18 and 70 were interviewed face-to-face by trained interviewers in each household. The data of this study, thus, comprised of surveys from 3 provinces, 4 counties, 16 townships, 64 villages, 1769 households, and 2847 participants. All participants were asked to provide written informed consent. The individual response rate was 100% (Appendix A Table A1).

The sociodemographic information of the respondents, including gender, age, marital status, etc., was collected. The abbreviated DRM questionnaire was used to assess rural residents’ daily activities and their affective SWB, with recall time limited to 15 min. It has been used and validated in the SAGE study of WHO [34,35] (available at: http://www.who.int/healthinfo/systems/GenericIndividualQ.pdf, accessed on 10 May 2020). For example, research by Miret et al. [35] compared SWB results from the abbreviated version of the DRM with those from the original long version among 1560 adults in Jodhpur, India, as part of the SAGE study. This study validated the use of the abbreviated version of the DRM aggregated over the population, which combined the morning, afternoon, and evening sets, and provided a profile similar to the original long version in the evaluation of affective state, with adequate test–retest properties tested one week apart. Ayuso-Mateos et al. [34], further, validated the same approach in a representative sample from seven countries, including China, Ghana, India, Mexico, Russia, South Africa, and Spain, and suggested that the abbreviated DRM is a useful tool for multi-country evaluation of affective SWB with adequate psychometric properties regarding reliability and construct validity in all countries (with composite reliability coefficients ranging from 0.77–0.91 for negative affect, and from 0.70–0.89 for positive affect; and with the goodness-of-fit indices CFI and TLI values higher than 0.98; RMSEA values from 0.026–0.074). In the present study, following the administration guideline of the abbreviated DRM questionnaire in SAGE study, instead of asking all participants to recall the entire preceding day starting from when they wake up, as in the original DRM version [17], the abbreviated version of the DRM randomly assigned participants to four different sets (A, B, C, and D) to reduce the investigation time [34]. In sets A, B, and C, Chinese rural residents reconstructed only a portion of their previous day’s activities from the morning when they wake up, from the afternoon when they have lunch, and from the evening when they have dinner, respectively, and responded to questions about each episode, including the type of the activity (e.g., eating, shopping), the time spent on each activity, interacting partners (e.g., alone, with a spouse), the friendliness you felt towards the interacting partners (e.g., very friendly, a little irritated), and seven affective feelings they experienced about each activity: Worried, rushed, irritated/angry, depressed, tense/stressed, calm/relaxed, and enjoying, which were reported on a 3-point scale (1 = not at all, 2 = a little, and 3 = very much). Participants kept reconstructing episodes until they arrived at the activity “went to sleep for the night”, or when 15 min of interview time have elapsed in a continuous activity-by-activity manner during the administration of sets A, B, and C [33]. Regarding set D, it required participants to recall various information in three parts of the day (morning, afternoon, and evening) together instead of activity by activity [18], so it was not included in the present analysis. In this study, the distribution of three sets (A, B, and C) of the abbreviated DRM questionnaire across counties showed no significant difference (X^2^ = 2.26, *p* = 0.894 > 0.05) (Appendix A Table A2). The questions of the abbreviated DRM questionnaire were translated from English into Chinese following the WHO’s translation guidelines for assessment instruments (available at: https://www.who.int/substance_abuse/research_tools/translation/en/, accessed on 10 May 2020).

### 2.2. Variables

#### 2.2.1. Outcome: Affect

In this study, three affective SWB indicators were studied: PoA, NeA, and net affect. PoA and NeA were defined as the average of the scores given to the two positive feelings (calm/relaxed, and enjoying), and the average of those given to the five negative feelings (worried, rushed, irritated/angry, depressed, and tense/stressed), respectively. Responses to each question had values between 1 to 3, with higher values indicating greater magnitude of PoA and NeA [18]. Net affect is a global measure that combines both positive and negative mood states, which was defined as the PoA minus the NeA, and it ranged between −2 and +2. Higher values of net affect represented higher affective SWB [9]. As a supplement, we also analyzed the diurnal rhythms of seven specific emotions to check if the diurnal rhythms were universal across feelings or driven only by certain emotions (results were provided in Appendix A).

#### 2.2.2. Time of Day: The Occurrence of Activities

In order to detect the diurnal rhythms of affect, we followed the rules of Stone et al. [6] and Lee et al. [18] to create the time of day (ToD) variable to represent the occurrence of activities. First of all, the time spent on each activity was used to compute the start and end times of the activities. For the first activity after waking up, the start time was recorded in the questionnaire by asking participants, *“At what time did this activity begin?”*, and the end time was calculated as the start time plus the time spent on it. Then, the start time of the second activity was the end time of the first activity, and the end time of this activity were computed as the start time plus the time spent on this activity. The start and end times of the other activities were calculated in a similar manner. Next, each activity’s midpoint time was calculated (the average of start and end times of each activity) to represent when the activity occurred. Subsequently, the ToD variable was generated in which the midpoints of activities were categorized into 20 one-hour blocks (e.g., 7:00–7:59). ToD was treated as a categorical variable in the present study.

#### 2.2.3. Social Environmental Factors: Activity Type and the Quality of Social Interaction

For two social environmental factors, activity type included working, subsistence farming, preparing food, doing housework, watching children, shopping, commuting, rest (includes tea/coffee break), chatting with someone, playing (includes cards/mahjong), reading, watching TV, exercising or leisurely walk, other leisurely activity, grooming or bathing (self), eating, other activity. We adopted the approach of Lee et al. [18] to measure the quality of social interaction, using three adjectives “alone”, “friendly”, and “irritating”. “Alone” represented activities being experienced alone. “Friendly” and “irritating” meant activities accompanied by friendly interacting partners and irritating interacting partners, respectively.

#### 2.2.4. Covariates

Covariates involved in the modes were measured as follows:

(1) Four counties: Anyue, Linquan, Caoxian, and Chiping;
(2) Three sets of DRM questionnaire: Morning set, afternoon set, and evening set;
(3) Sociodemographic factors: Gender (male, female); age and age^2^ (from 18–70, and treated as continuous variable in models); the highest education level completed (no formal education, primary school, middle school, and above); marital status (married, other); occupation (farmer and other (this category represented participants who had multiple occupations not only as farmers, but also as workers, businessman, teachers, and village cadres, etc.), farmer, non-farmer (this category represented participants who were not farmers, such as workers, village cadres, businessman, teachers, students, and the unemployed, etc.)); self-perceived health (very well, well, general, bad, very bad). The wealth of rural residents was evaluated with the International Wealth Index (IWI) [56]. IWI was treated as a continuous variable in models, and the larger the value of IWI, the richer people are.

### 2.3. Statistical Analysis

Multilevel models were used to predict the diurnal patterns of affect among Chinese rural residents. In the present study, the measurement of affective SWB at different time points provided clustered data, with repeated measures of the outcomes at Level 1 clustered within individuals at Level 2. To counteract the unbalanced nature of the dataset, with data missing for some individuals at a specific survey time, we used a multilevel model to analyze change over time, so that the number of Level 1 units clustered at Level 2 does not need to be equal [57,58,59,60]. In the present study, we constructed two multilevel models to detect the diurnal rhythms of affect among rural Chinese. Model 1 depicted the diurnal patterns of affect by controlling for county, sets of DRM questionnaire, and sociodemographic factors of rural residents. The second model examined to what extent diurnal patterns of affect remained after further controlling for two social environmental factors—activity type and the quality of social interaction on the basis of model 1. It might be expected that the diurnal trajectories of affect might vary from individual to individual, so ToD was also added in a random slope to test the fit of models. However, adding in a random slope for ToD did not improve the fit of the model, so in the present study, ToD was used as a categorical fixed parameter in multilevel models. The models were specified as follows:

- Model 1:
  (1) $$Y_{tj}{=B}_{0tj}+B_{1}\left({\mathrm{county}}_{j}\right)+B_{2}\left({\mathrm{sets}}_{j}\right)+B_{3}\left({\mathrm{sociodemographic}\;\mathrm{factors}}_{j}\right)+B_{4}({\mathrm{ToD}}_{tj})$$
- Model 2:
  (2) $$Y_{tj}=B_{0tj}+B_{1}\left({\mathrm{county}}_{j}\right)+B_{2}\left({\mathrm{sets}}_{j}\right)+B_{3}\left({\mathrm{sociodemographic}\;\mathrm{factors}}_{j}\right)+B_{4}({\mathrm{ToD}}_{tj})+B_{5}({\mathrm{activity}}_{tj})+B_{6}({\mathrm{quality}}_{tj})$$
- For two models:
  (3) $$B_{0tj}=B_{0}+u_{0j}+e_{0j}$$
  where t (t = 1,…,$T_{j}$) indicates the Level 1 units (ToD) within j (j = 1,…,n) Level 2 units (individuals); $Y_{\mathrm{tj}}$ is the affect scores at time t of an individual j; $B_{0}$ is the fixed intercept; $B_{1}$ is a coefficient for county; $B_{2}$ is a coefficient for three sets of DRM questionnaire; $B_{3}$ is a coefficient for participants’ sociodemographic factors; $B_{4}$ is a coefficient for the ToD; $B_{5}$ is a coefficient for the activity type; $B_{6}$ is a coefficient for the quality of social interaction. $u_{0j}$ and $e_{0tj}$ are the individual level residuals and ToD level residuals, and are assumed to be normally distributed.

## 3. Results

### 3.1. Demographic Information

Table 1 showed the demographic information of participants. Of the 2847 Chinese rural residents surveyed, there were more women (57.18%) than men (42.82%). The average age was 46 (SD = 12.47), and the vast majority were married (91.89%). Almost one-third (32.81%) did not receive any formal education. Farmers (69.86%) accounted for two-thirds of all respondents. The mean value of IWI was 44.86 (SD = 12.60). Half of the rural residents (50.02%) rated their health status “well” and above.

### 3.2. Activity Information

#### 3.2.1. Activity and Affective SWB

Table 2 summarizes the information about each activity and mean affect ratings. We excluded the activities “went to sleep for the night”, as participants were not asked to report time duration and their feelings during sleeping. A total of 14,746 activities were recalled, and the average number was 5.2 (SD = 2.47). The mean time participants reported on the previous day was 6.5 h (SD = 3.97). The three most commonly recalled activities were eating (30.12%), subsistence farming (11.64%), and preparing food (10.50%). Chinese rural residents spent the longest time on working (3 h), followed by subsistence farming (2.61 h), playing (includes cards/mahjong) (2.33 h), and watching children (2.32 h).

With respect to SWB ratings, higher PoA ratings were found when playing (includes cards/mahjong) (2.63), chatting with someone (2.60), watching TV (2.59), and exercising or leisurely walk (2.54), whereas lower levels of PoA were observed for working (1.86), and subsistence farming (1.94). On the contrary, working (1.32), watching children (1.32), and subsistence farming (1.28) were associated with higher levels of NeA. Chatting with someone (1.06), playing (includes cards/mahjong) (1.07), and watching TV (1.04) were associated with lower NeA among Chinese rural dwellers. Higher net affect ratings were found in chatting with someone (1.54), playing (includes cards/mahjong) (1.57), watching TV (1.55), and exercising or leisurely walk (1.43), which suggested a stronger sense of happiness during these activities. However, working (0.55), subsistence farming (0.66), watching children (0.85), and doing some household chores, such as preparing food (0.86), made rural residents relatively unhappy with lower net affect scores (Table 2).

**Figure 1:**
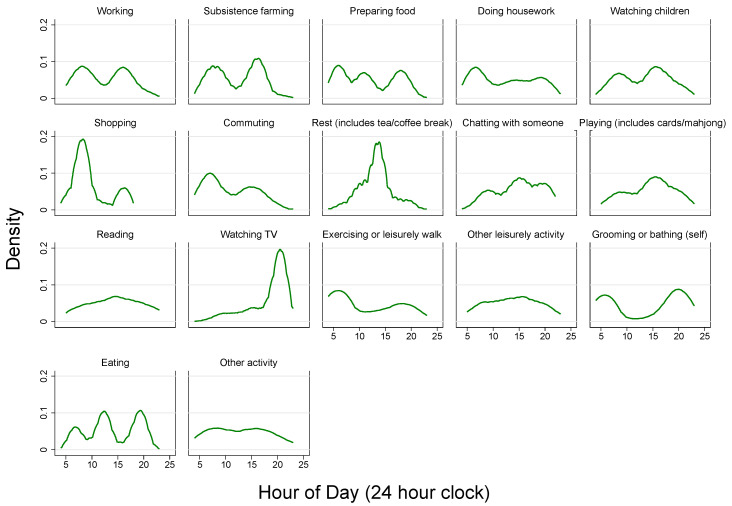
Kernel density plots of time of day by activities.

We created kernel density plots of ToD by different activities to observe the occurrence time of each activity throughout the day (Figure 1). The plots showed that some low-SWB activities, such as working, subsistence farming, watching children, shopping, and commuting, occurred more often in the mid-morning and (or) mid-afternoon, while some high-SWB activities, such as rest, chatting with someone, playing (includes cards/mahjong), and watching TV usually took place around lunchtime or in the evening. Exercising or leisurely walk and grooming or bathing (self) showed similar ToD trends with the occurrence time in the early morning and in the evening. There were three peaks in the occurrence time of preparing food and eating (in the early morning, at around noon, and in the evening). For reading, leisurely activities, and other activities, these tended to happen in the afternoon.

#### 3.2.2. The Quality of Social Interaction

The vast majority of activities were done with friendly interacting partners (54.38%). More than 40% of activities were carried out alone by participants. Only 1.13% of rural residents felt irritated towards their interacting partners (Table 3).

### 3.3. Model of Diurnal Rhythms of Affect

In this part, we modeled the diurnal rhythms for Chinese rural residents’ SWB indicators, including PoA, NeA, and net affect, by building two multilevel models. The first model (model 1) examined the relationship between affect and ToD by controlling for county, sets of DRM questionnaire, and sociodemographic factors of rural residents. The second model (model 2) further controlled for two social environmental factors—activity type and the quality of social interaction based on model 1, to examine to which extent diurnal patterns of affect remains when the influence of social environmental factors was removed. Tests of diurnal rhythms of seven specific emotions were also conducted as a supplement and presented in Appendix A.

#### 3.3.1. Diurnal Rhythms of Affect from Model 1

**Figure 2:**
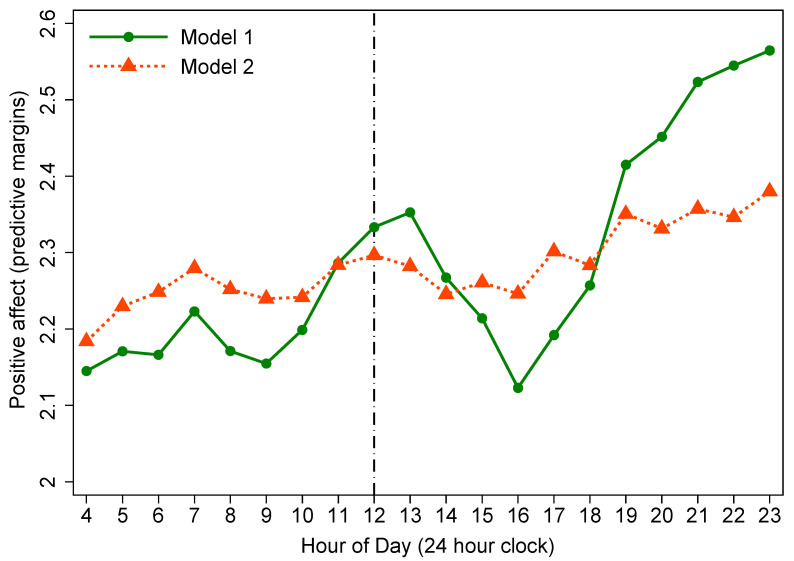
Diurnal rhythm of positive affect (reference group: 4:00).

**Figure 3:**
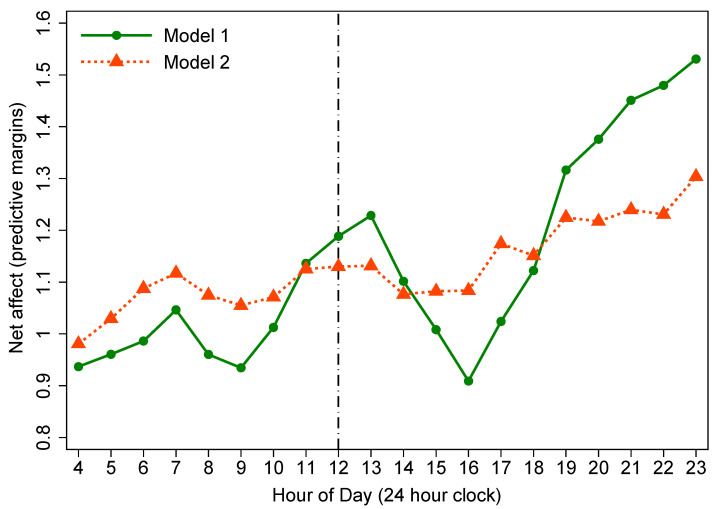
Diurnal rhythm of net affect (reference group: 4:00).

**Figure 4:**
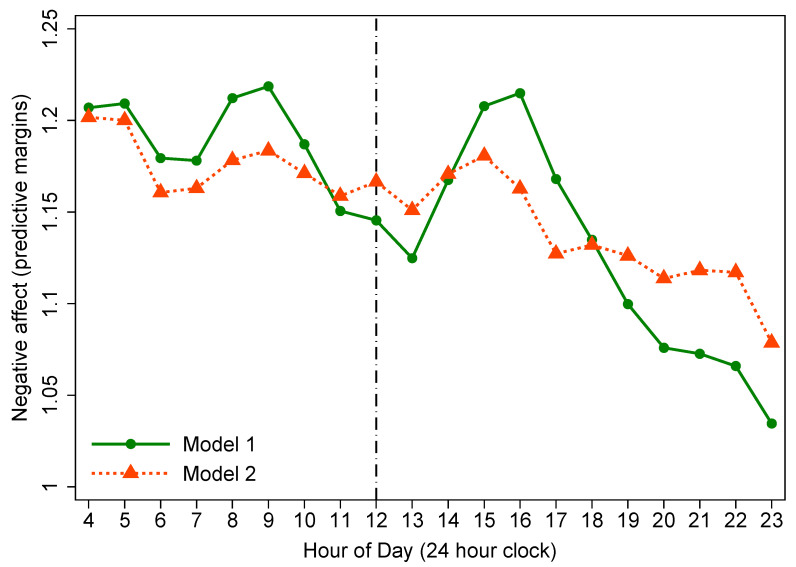
Diurnal rhythm of negative affect (reference group: 4:00).

Model 1 in Table 4 shows that the diurnal rhythms of PoA and net affect generally increased throughout the day, whereas NeA tended to decrease as the day passed. In order to observe the changing trend of each SWB indicator throughout the day, we made plots to graphically present the predicted diurnal rhythms of affect at each hour of the day. Double-peak diurnal patterns were observed in PoA and net affect. The magnitude of PoA and net affect of rural residents showed an incremental trend from about 9:00 that lasted to the first peak at 13:00 (from 11:00–13:00, all *p* < 0.05). Afterward, it gradually eased back to a lower level in the afternoon, and then began to rebound from approximately 16:00, reaching the second peak at bedtime (from 19:00–23:00, all *p* < 0.001) (Figure 2 and Figure 3). However, for NeA, a double-trough diurnal pattern was evident in the plot. Specifically, from waking up in the morning, the magnitude of NeA during the day tended to decrease roughly from 9:00, and reached its first nadir at 13:00 (from 12:00–13:00, all *p* < 0.05). Then it rose back to the morning level until 16:00. From 16:00 and onwards, a second decline in NeA appeared, and its second lowest point was at 23:00 (from 18:00–23:00, all *p* < 0.05) (Figure 4).

#### 3.3.2. Diurnal Rhythms of Affect from Model 2

Earlier studies demonstrated that social environmental factors—activity type and the quality of social interaction, significantly influenced the diurnal rhythms of affect [6,18]. Therefore, we further controlled for social environmental factors in model 2 (Table 4). Model 2 results show that in terms of PoA and NeA, the estimated coefficients for ToD around noon were no longer statistically significant. For net affect, the *p* values of the estimates at 12:00 and 13:00 were borderline significant (*p* = 0.047 and 0.046, respectively).

From Figure 2, Figure 3 and Figure 4, we observed that the effects of ToD on SWB indicators have been reduced when comparing the estimates from model 1 to model 2, with all diurnal patterns of affect flattening to some extent in model 2 after taking into account social environmental factors in the models. Specifically, for three SWB indicators, the lunchtime peaks in positive affect and net affect (and trough in negative affect) were eliminated entirely, indicating an important contribution of social environmental factors to diurnal rhythms of affect.

Additionally, from the results of activity type in Table 4, it is evident that compared with working, activities that were associated positively with PoA and net affect, were negatively associated with NeA. With respect to the quality of social interaction, compared with “being alone”, activities with friendly partners were significantly associated with higher PoA and net affect, whereas irritating interacting partners gave rise to significantly lower PoA and net affect, suggesting a detrimental impact on affective SWB. There was no clear difference in NeA between engaging in activities with friendly partners and doing activities alone, but activities with irritating partners had a significantly positive association with NeA.

**Figure A1:**
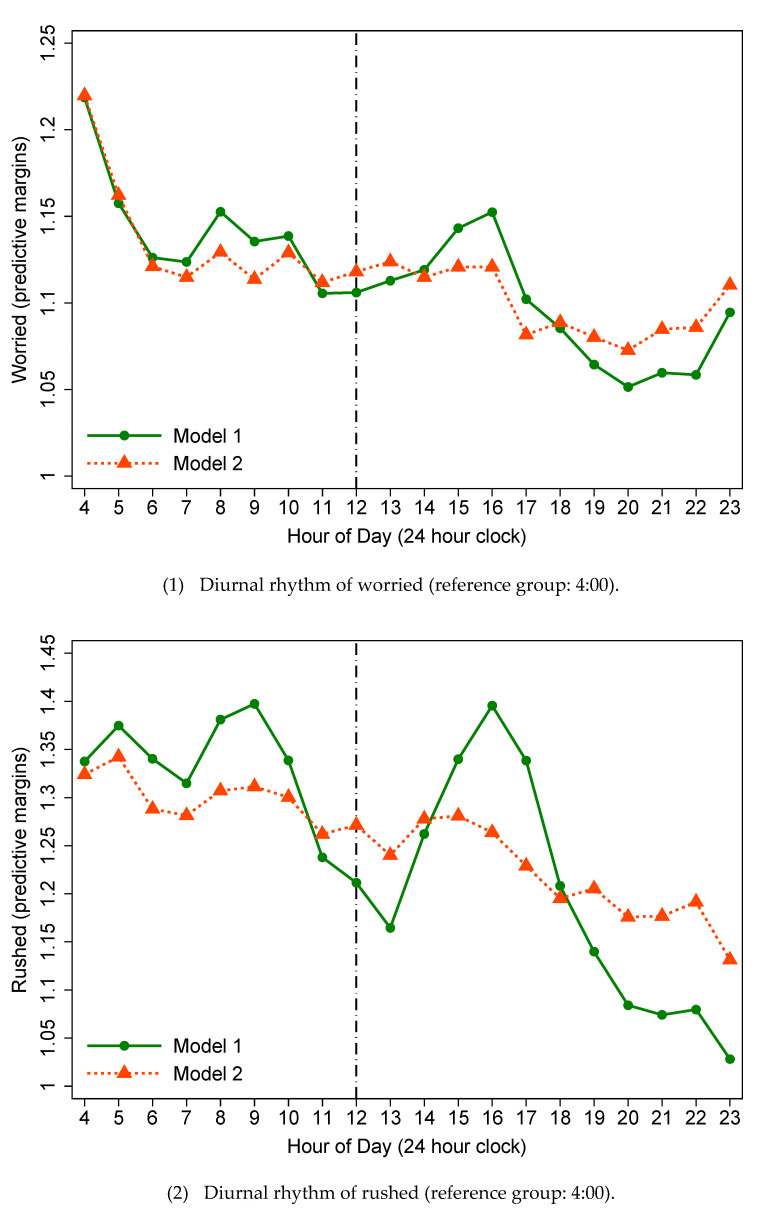

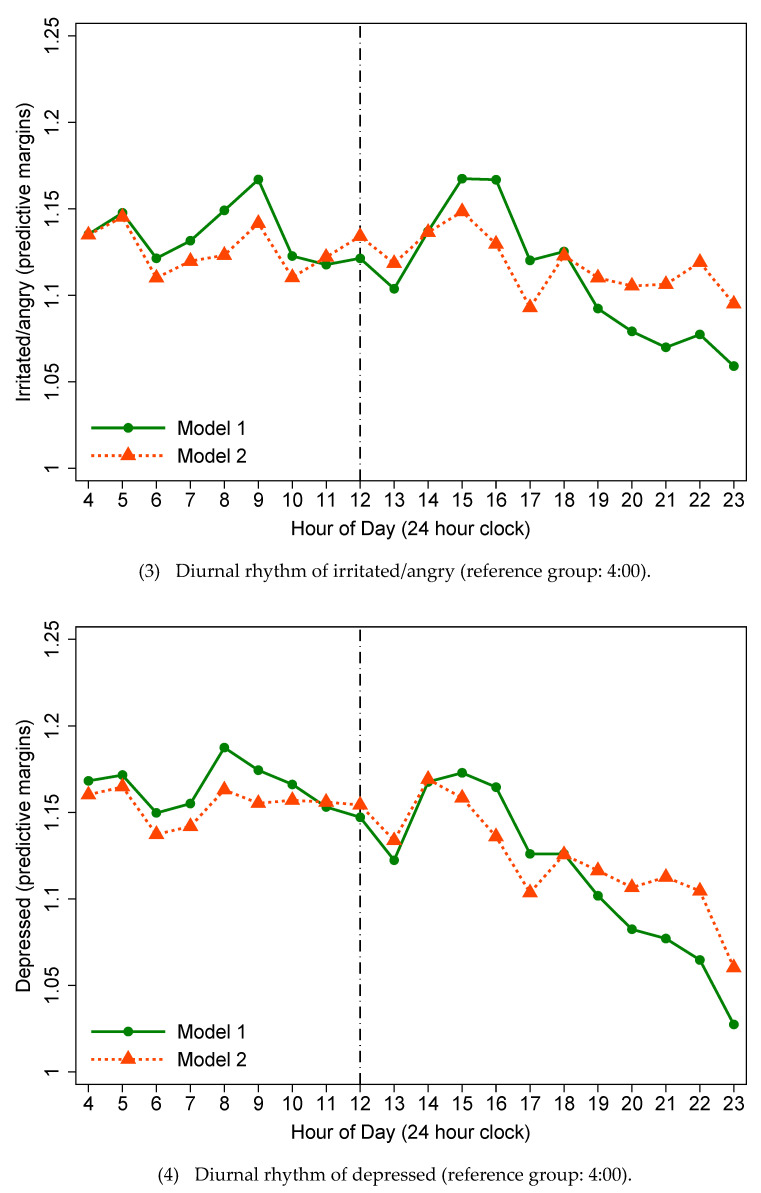

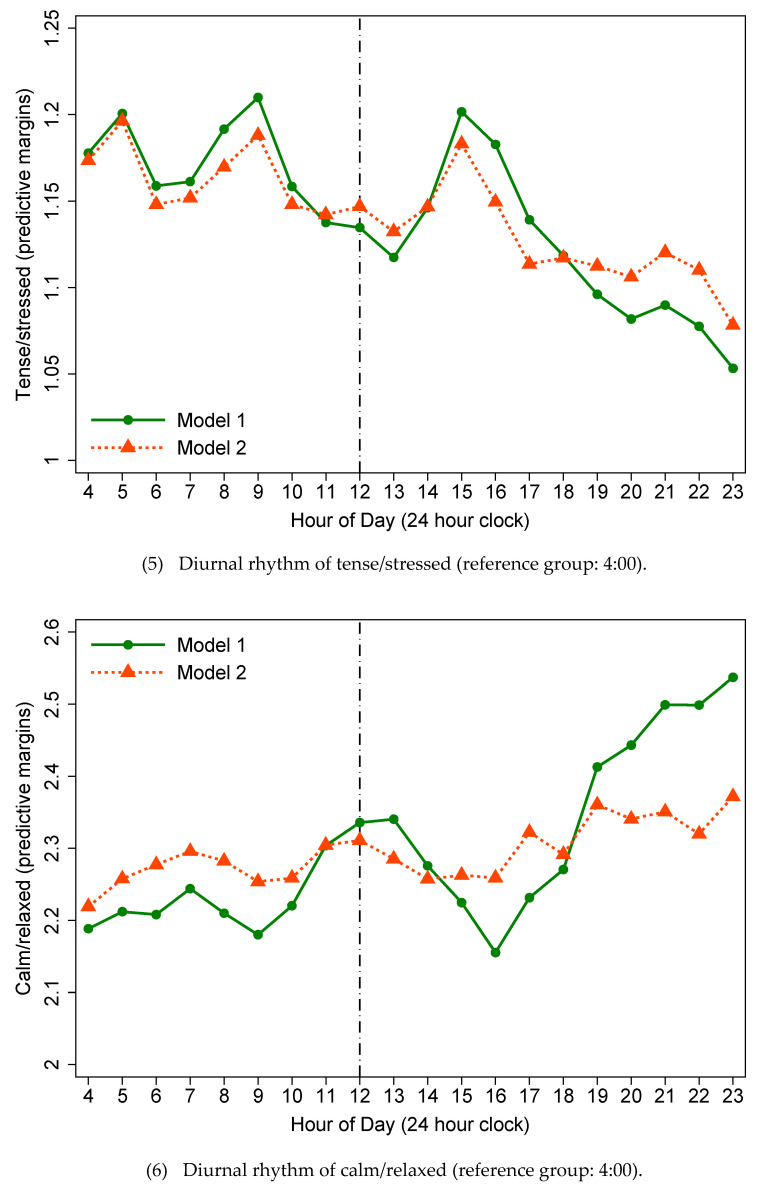

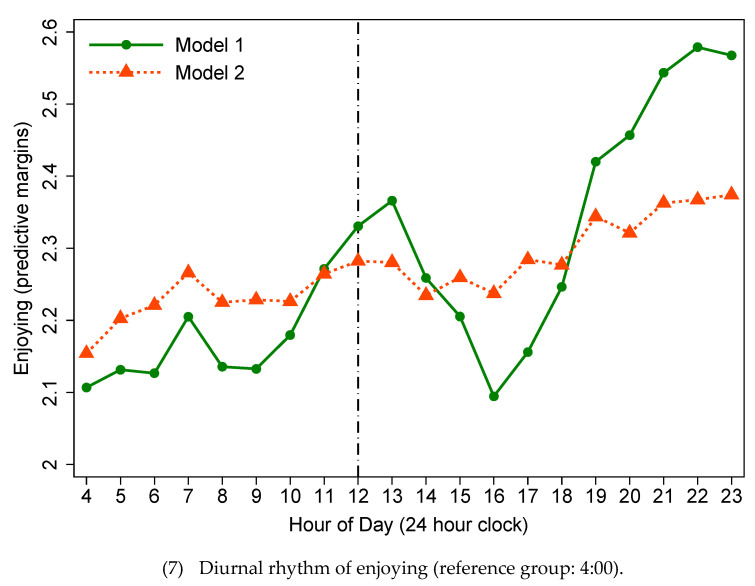
Diurnal rhythms of seven emotions (reference group: 4:00).

We analyzed the diurnal rhythms of seven specific emotions as a supplement to the analysis presented above. Results of regression models (models 1 and 2) (Table A3) and the resulting diurnal rhythm plots (Figure A1) for all emotions were presented in Appendix A. Based on model 1, we found that for two positive emotions (calm/relaxed and enjoying), both of them generally increased throughout the day with consistent bimodal diurnal patterns. Peaks at lunchtime and in the evening were observed (for calm/relaxed, from 12:00–13:00 and from 19:00–23:00, all *p* < 0.05; for enjoying, from 11:00–13:00 and from 19:00–23:00, all *p* < 0.05). However, for the five negative emotions, more complicated diurnal changing trends were observed. First, for the general changing trend with ToD, all negative emotions (worried, rushed, depressed, tense/stressed), except for irritated/angry (no significant diurnal rhythm existed throughout the day), exhibited a significant downward trend as the day passed. Second, for the hourly changing trend, similar double-trough diurnal rhythms with nadirs, respectively, at lunchtime and in the evening were observed for worried and rushed (for worried, from 9:00–14:00 and from 17:00–23:00, all *p* < 0.05; for rushed, from 12:00–13:00 and from 18:00–23:00, all *p* < 0.05). Nevertheless, worried had a relatively distinct hourly diurnal rhythm. Specifically, there was a significant drop in magnitude from 4:00–7:00, then it remained roughly stable at a lower level until 16:00 with a nadir occurring at 11:00. Afterward, a second noticeable decline lasted till 20:00, followed by a slight rebound. For feeling depressed and tense/stressed, these were weakly associated with hour of the day with only one significant trough observed in the evening (from 20:00–23:00, all *p* < 0.05). It is worth noting that when we further considered the activity type and the quality of social interaction in model 2, the diurnal patterns of all seven emotions flattened to some extent.

## 4. Discussion

The present study examined diurnal rhythms of affective SWB among Chinese rural residents. When models only controlled for county, sets of DRM questionnaire and sociodemographic factors, PoA and net affect generally increased in magnitude over the day, whereas NeA showed an overall decrease as the day passed. Bimodal patterns with peaks at noon and in the evening were detected for PoA and net affect. Correspondingly, a diurnal pattern, with troughs, respectively, at lunchtime and in the evening, was identified in NeA. However, when the influences of two environmental factors—activity type and the quality of social interaction were taken into account, a substantial flattening of the diurnal trajectories occurred for all SWB indicators with the lunchtime peak in PoA and net affect, and trough in NeA disappearing entirely, indicating the important contribution of environmental factors to diurnal rhythms of affect among rural Chinese residents.

With regard to the overall diurnal rhythms of SWB indicators, we replicated the results from Western studies that PoA and net affect show a tendency to increase over the course of the day, while NeA declines throughout the day [6,17,29,34]. For the specific hourly diurnal patterns of affect, in the present study, the magnitude of PoA and net affect of rural Chinese tended to increase from about 9:00 and lasted till the first peak at 13:00. Afterward, it gradually eased back to a lower level in the afternoon, and then began to rebound from approximately 16:00, reaching the second peak at bedtime. Research by Ayuso-Mateos et al. [34], as one of the WHO’s SAGE studies, covering seven diverse countries (China, Ghana, India, Mexico, Russia, South Africa, and Spain) investigated 14,811 adults aged 18-plus years from China and illustrated that in China, PoA and net affect improved as the day passed with peaks occurring at about 13:00 and 21:00, respectively. Stone et al. [6] detected that positive affect showed bimodal patterns with peaks at noon and in the evening by investigating 909 American working women. We consistently confirmed these diurnal patterns among rural Chinese adults. Regarding NeA, we observed that from waking up in the morning, Chinese rural residents’ negative affect level remained stable in most of the morning, then showed a significant descending trend at about 9:00, and reached its first nadir at 13:00. Subsequently, it gradually rose back to the morning level in the afternoon. From 16:00 and onwards, a second significant decrease occurred with its second lowest point at bedtime. Ayuso-Mateos et al. [34] reported in their multi-country evaluation of affective SWB that NeA was most pronounced in the morning and tended to decrease throughout the day with two nadirs appearing at about 13:00 and 21:00. Our study consistently replicated their results in a rural population. Stone and colleagues [6] reported bimodal diurnal patterns in NeA with peaks at about 10 am and then at 4 or 5 pm. Nevertheless, a double-trough diurnal pattern, instead of double-peak rhythm, was identified in NeA of the present study.

There are several reasons that might explain the diurnal rhythms of affect reported in the present study. First, PoA and net affect showed sizable jumps around noon and in the evening, while NeA showed corresponding considerable declines. This is possibly linked to specific activities such as lunchtime, when people can enjoy their lunch and have some rest, which provides a respite from the demands of the work (or farming, household chores). And evening time represents the end of a whole day’s work, when rural residents can fully enjoy their time with their families and engage in some pleasurable activities (e.g., watching TV, and chatting with someone), reducing negative affect and increasing positive affect [6]. Second, the different diurnal rhythms found in NeA between our study and Stone et al. [6] research might be explained by a different target population (e.g., rural Chinese versus American working women), the nature of the lifestyles of people in the different countries, and the kind of activities that people engage in [33].

Our results also showed that social environmental factors played a vital role in shaping the diurnal rhythms of affect among Chinese rural residents. As there is little evidence on the role of social environmental factors in explaining the diurnal patterns of affect, we compared our results with findings from Stone et al. [6]. In the present study, different types of activity might influence affect at different hours of the day. For example, results of the present study indicated that several high-SWB activities, such as eating, rest (includes tea/coffee break), chatting with someone, playing (includes cards/mahjong), and watching TV, were significantly associated with increased PoA and decreased NeA (Table 4). Figure 1 graphically illustrated that these pleasurable activities always happened around noon and evening time, contributing to the considerable elevation of PoA and net affect and drop of NeA at the corresponding time. This is consistent with findings from Stone et al. [6]. Despite different target population between these two studies (rural Chinese versus American working women), we drew a consistent conclusion, adding up-to-date evidence to the existing Global South-focused SWB literature. We found that the quality of social interaction also exerted an influence on the magnitude of affect across the day. Compared with activities done alone, activities accompanied by friendly interacting partners were significantly associated with higher PoA and net affect scores. Furthermore, we showed that being alone was better than activities with irritating interacting partners, which were significantly associated with decreased PoA and net affect, and increased NeA (Table 4). Our results consistently confirmed the findings from the research of Lee et al. [18].

As a supplement, we also analyzed the diurnal rhythms of seven specific emotions. Most of them (for both positive and negative emotions) exhibited consistent diurnal patterns with PoA and net affect (or NeA), except for worried and irritated/angry. For the diurnal rhythm of worried, it plummeted in the early morning, then it remained roughly stable at a lower level for most of the day time. A second significant decline took place from 16:00, followed by a slight rebound at bedtime. Stone et al. [6] reported a decreasing diurnal rhythm of worried with peaks found at mid-morning and mid-afternoon among American working women. Our results were different from theirs. The possible reasons might be attributed to different target populations and different cultural attitudes. One possibility is that Chinese rural residents may feel more competent with their progress of work during the day, and the resolution of challenges from work may lead to the first decline in worry. As the work day closes, people finish the heavy work activities and start to enjoy some pleasurable and relaxing activities, such as watching TV and chatting with someone, which may give rise to the second significant drop of worry. The slight increase in worry before bedtime may reflect residents’ concerns for the next day’s work. Regarding irritated/angry, no significant diurnal rhythm was found in the present study, while Stone et al. [6] illustrated a weak association between being angry and the hour of the day. The reason for this phenomenon was unclear, but we could still observe a weak diurnal pattern of irritated/angry throughout the day, with two troughs occurring at lunchtime and in the evening (Figure A1). Future research with a larger sample size of participants can be carried out to gain a deeper understanding of the lack of a diurnal rhythm in irritated/angry.

Results of the present study suggest that diurnal rhythms of affect were able to reflect the situation of social environmental factors, and conversely, they could change in response to stimulation from the environment. Given the evidence that increased negative affect is associated with increased health risks, such as coronary heart disease, disability, and other chronic disorders [13], while frequent high level of positive affect has been proved to protect people against poor health [14], the evidence of the present research is important and helpful for designing further intervention studies to prevent diseases and encourage health-promoting behaviors. For example, interventions on some social environmental factors, such as organizing pleasant activities and creating a friendly environment (e.g., companionship of friendly partners), could contribute to the increase in positive affect and decline in negative affect in daily life. In addition, enhancing positive emotions and reducing negative emotions can bring more comprehensive benefits to our daily lives. The broaden-and-build theory developed by Barbara L. Fredrickson [61] suggests that positive emotions, such as joy, interest, contentment, and love, can promote the discovery of novel and creative actions, ideas, and social bonds by broadening individual’s momentary thought–action repertoire (providing the individual with a wider range of thoughts and actions to choose to draw upon), which in turn build enduring personal resources over time, ranging from physical and intellectual resources, to social and psychological resources. Evidence from the present study that both PoA and NeA could change in response to stimulation from the environment might be helpful in cultivating positive emotions (such as by optimizing social environmental factors to improve PoA) in people’s own lives and in the lives of those around them, thereby not only making them feel good in the moment, but also transforming people for the better and setting them on paths toward flourishing and healthy longevity.

Several limitations of the present study should be considered. First, the data were collected from Chinese rural residents in four counties of three provinces, so the diurnal rhythms identified in the present study might not be able to be generalized to all rural Chinese or other populations. Second, in addition to the environmental factors, the diurnal rhythms of affect may be also influenced by diurnal endogenous factors, such as cortisol, and growth hormone [19], but the present research was unable to take account of these factors owing to the lack of relevant information in the dataset. Additionally, although studies have indicated that DRM could successfully reduce recall bias when evaluating momentary emotions by showing comparable results with ESM and adequate psychometric properties, others have argued that as the DRM relies on retrospective self-reports, this could possibly produce some general methodological problems. Individuals with higher levels of cognitive ability may be better able to reconstruct their day, whereas participants of lower ability may forget what they were doing or how they felt about the activity [62]. For example, respondents with poor memory assigned to the morning set might not be able to recall all the activities experienced in the morning of the previous day within 15 min, while some others might be able to recall all the activities as they experienced during the whole day. Meanwhile, the time taken by interviewers to collect these data might also affect the recording of these activities with limited recall time. These features of the DRM survey design might cause selection bias in constructing diurnal rhythms of affect throughout the previous day. Furthermore, endogenous personality traits, such as extraversion or optimism, might also introduce bias. Research by Newton et al. [63] conducted an examination on extraversion associated with activity participation and activity-affective experience, revealing that extraverts were more likely to socialize and experienced higher socializing-related positive affect. Extraverted people might be more likely to engage in some high-SWB activities (e.g., chatting with someone) instead of some personal leisurely activities (e.g., reading), and this trait might result in higher PoA and lower NeA. Therefore, the disappearance of lunchtime peaks in PoA and net affect and trough in NeA after controlling for activity types and the quality of social interaction might be explained by endogenous personality traits that influence the choice of social activities. However, we also argued that this might be the case, due to the social ordering of time. For example, at lunchtime, everyone needs to eat, regardless of their personality types. We found that eating was a more enjoyable activity than most other activities during the day. Similarly, in the evening, social time rhythms in rural China dictate that you are unable to work in the field (for example, because there is not enough light) but relax, and people tend to select activities that are more enjoyable. However, due to the lack of relevant data and information in the dataset, we were unable to analyze the confounding effect of personality traits. Further research may take this into account when reporting diurnal rhythms of affect. Finally, the results of the present study were compared with findings from Stone et al. [6] research, but owing to different measurement scales of affect (six-point scales versus three-point scales), the comparability of results needs to be treated with cautions [29].

## 5. Conclusions

The present research is the first, to our knowledge, to detect the diurnal rhythms of affective SWB using DRM in rural China. PoA and net affect generally increased in magnitude over the course of the day with two peaks around noon and in the evening, respectively. Conversely, NeA showed an overall decline as the day passed, with two troughs occurring at lunchtime and in the evening. However, after taking into account the effects of two social environmental factors—activity type and the quality of social interaction, diurnal patterns flattened considerably with the lunchtime peak in PoA and net affect (and trough in NeA) eliminated entirely, indicating social environmental factors had a significant effect on diurnal rhythms of affect. Results of the present study suggested that diurnal rhythms of affect could change in response to stimulation from the environment. Given that diurnal rhythms of affect are important for people’s health and behavior, this study has the potential to contribute to relevant interventions, such as improving social environmental factors, that could enhance the quality of life of rural Chinese adults.

## Figures and Tables

**Table 1:** Demographic information of participants.

Participants	*N* (%)
Gender	
Male	1219 (42.82)
Female	1628 (57.18)
Highest education level completed ^#^	
No formal education	934 (32.81)
Primary school	851 (29.89)
Middle school and above	1058 (37.16)
Marital status	
Married	2616 (91.89)
Other	231 (8.11)
Occupation ^#^	
Farmer	1989 (69.86)
Farmer and other	419 (14.72)
Non-farmer	437 (15.35)
Self-perceived health status ^#^	
Very well	408 (14.33)
Well	1016 (35.69)
General	719 (25.25)
Bad	587 (20.62)
Very bad	100 (3.51)
Total sample size	2847 (100)

Note: ^#^ Missing values were identified in these variables.

**Table 2:** Information of each type of activity and mean affect ratings.

Activity	*N* (%)	Mean Time in Hours (SD)	Mean Affect Ratings (SD)		
			Positive Affect	Negative Affect	Net Affect
Working	466 (3.16)	3.00 (1.80)	1.86 (0.67)	1.32 (0.40)	0.55 (0.93)
Subsistence farming	1716 (11.64)	2.61 (1.52)	1.94 (0.69)	1.28 (0.38)	0.66 (0.94)
Preparing food	1549 (10.50)	0.69 (0.34)	2.05 (0.67)	1.19 (0.33)	0.86 (0.86)
Doing housework	1048 (7.11)	0.90 (0.82)	2.07 (0.65)	1.21 (0.33)	0.86 (0.85)
Watching children	330 (2.24)	2.32 (1.55)	2.17 (0.69)	1.32 (0.41)	0.85 (0.98)
Shopping	55 (0.37)	2.04 (1.21)	2.25 (0.78)	1.14 (0.31)	1.11 (0.95)
Commuting ^#^	241 (1.63)	0.69 (0.81)	2.13 (0.65)	1.13 (0.23)	0.99 (0.76)
Rest (includes tea/coffee break)	1488 (10.09)	1.62 (1.05)	2.50 (0.63)	1.08 (0.22)	1.42 (0.75)
Chatting with someone	583 (3.95)	1.73 (1.11)	2.60 (0.56)	1.06 (0.18)	1.54 (0.66)
Playing (includes cards/mahjong)	240 (1.63)	2.33 (1.49)	2.63 (0.54)	1.07 (0.22)	1.57 (0.66)
Reading	68 (0.46)	1.54 (1.15)	2.37 (0.66)	1.18 (0.33)	1.19 (0.89)
Watching TV	1472 (9.98)	1.59 (0.94)	2.59 (0.54)	1.04 (0.16)	1.55 (0.61)
Exercising or leisurely walk	229 (1.55)	1.04 (0.80)	2.54 (0.61)	1.11 (0.24)	1.43 (0.76)
Other leisurely activity ^@^	100 (0.68)	1.67 (1.31)	2.52 (0.61)	1.11 (0.30)	1.41 (0.83)
Grooming or bathing (self)	662 (4.49)	0.34 (0.23)	2.46 (0.59)	1.08 (0.21)	1.38 (0.70)
Eating	4442 (30.12)	0.57 (0.29)	2.39 (0.62)	1.11 (0.26)	1.28 (0.77)
Other activity *	57 (0.39)	1.87 (1.86)	1.79 (0.71)	1.45 (0.49)	0.34 (1.06)

Note: ^#^ Commuting combined walking somewhere, traveling by bicycle, and traveling by car/bus/train together. Commuting here broadly represents rural residents traveling between two places, not necessarily between home and workplace. ^@^ Listening to the radio was included in other leisurely activity, due to only a few responses. * Religious activity and providing care to someone were included in other activity, due to only a few responses.

**Table 3:** The quality of interaction.

The Quality of Interaction	*N* (%)
Alone	6161 (44.48)
Friendly	7532 (54.38)
Irritating	157 (1.13)

**Table 4:** Results of multilevel analyses of time of day (ToD) and SWB indicators.

Variable	Model 1			Model 2		
	Positive Affect	Negative Affect	Net Affect	Positive Affect	Negative Affect	Net Affect
County (Ref. Anyue) ^@^						
Linquan	0.0082	−0.01713	0.02486	−0.03421	−0.01157	−0.02252
Caoxian	0.02886	−0.00751	0.03603	0.02476	−0.01346	0.03814
Chiping	0.13146 ***	−0.04126 **	0.17208 ***	0.12433 ***	−0.04271 **	0.16688 ***
Sets of DRM questionnaire (Ref. Morning set)						
Afternoon set	0.01086	0.01425	−0.00439	−0.01009	0.02131	−0.03189
Evening set	−0.03187	0.03728 **	−0.06819	−0.03894	0.04145 **	−0.07894 *
Gender (Ref. Male)						
Female	−0.05138 *	0.02431 *	−0.07624 *	−0.01059	0.01544	−0.02623
Age	−0.01318 *	0.01098 ***	−0.02440 **	−0.00493	0.00896 **	−0.01413
Age^2^	0.00016 *	−0.00013 ***	0.00029 **	0.00007	−0.00011 ***	0.00018 *
Highest education level completed (Ref. No formal education)						
Primary school	0.08473 **	−0.02127	0.10510 **	0.08204 **	−0.02109	0.10195 **
Middle school and above	0.03014	−0.02560 *	0.05435	0.02658	−0.02519 *	0.05046
Marital status (Ref. Married)						
Other	0.0048	0.0304	−0.0259	0.00152	0.0357	−0.03465
Occupation (Ref. Farmer and other)						
Farmer	−0.02922	−0.01915	−0.00991	−0.03331	−0.02025	−0.01274
Non-farmer	−0.05956	−0.02267	−0.03752	−0.07026	−0.0228	−0.04778
Self-perceived health (Ref. Very well)						
Well	−0.14022 ***	0.03292 *	−0.17206 ***	−0.14214 ***	0.03268 *	−0.17397 ***
General	−0.18601 ***	0.04264 **	−0.22706 ***	−0.18227 ***	0.04083 **	−0.22197 ***
Bad	−0.22579 ***	0.09094 ***	−0.31513 ***	−0.23524 ***	0.09312 ***	−0.32723 ***
Very bad	−0.32886 ***	0.20882 ***	−0.53589 ***	−0.32646 ***	0.20636 ***	−0.53242 ***
IWI ^#^	0.00226 **	−0.00096 *	0.00324 **	0.00159	−0.00079 *	0.00240 *
Time of day (Ref. 4:00 (4:00–4:59))						
5:00 (5:00–5:59)	0.02584	0.00225	0.02371	0.04598	−0.00164	0.04865
6:00 (6:00–6:59)	0.02129	−0.02754	0.04931	0.06463	−0.04107	0.10686
7:00 (7:00–7:59)	0.0781	−0.02891	0.10955	0.09581	−0.03875	0.13642
8:00 (8:00–8:59)	0.02622	0.0052	0.02336	0.06814	−0.02343	0.09383
9:00 (9:00–9:59)	0.00979	0.01164	−0.00236	0.05588	−0.01818	0.07416
10:00 (10:00–10:59)	0.05384	−0.02	0.07567	0.05778	−0.03057	0.09026
11:00 (11:00–11:59)	0.14135 *	−0.05638	0.19933 *	0.09992	−0.04293	0.14448
12:00 (12:00–12:59)	0.18824 **	−0.06151 *	0.25161 **	0.11285	−0.03523	0.14907 *
13:00 (13:00–13:59)	0.20769 **	−0.08217 **	0.29175 **	0.09834	−0.05073	0.15068 *
14:00 (14:00–14:59)	0.12228	−0.03945	0.16471	0.0619	−0.03096	0.09559
15:00 (15:00–15:59)	0.06901	0.00084	0.07132	0.07697	−0.02105	0.10153
16:00 (16:00–16:59)	−0.02207	0.00793	−0.02784	0.06236	−0.03898	0.10298
17:00 (17:00–17:59)	0.047	−0.03891	0.08717	0.11786	−0.07448 **	0.19331 *
18:00 (18:00–18:59)	0.11205	−0.07212 *	0.18540 *	0.09948	−0.06970 *	0.17037 *
19:00 (19:00–19:59)	0.27006 ***	−0.10723 ***	0.37944 ***	0.16681 **	−0.07555 **	0.24393 **
20:00 (20:00–20:59)	0.30671 ***	−0.13110 ***	0.43893 ***	0.14767 *	−0.08801 **	0.23650 **
21:00 (21:00–21:59)	0.37842 ***	−0.13433 ***	0.51422 ***	0.17341 **	−0.08349 **	0.25878 **
22:00 (22:00–22:59)	0.39993 ***	−0.14102 ***	0.54299 ***	0.16256 *	−0.08462 **	0.24992 **
23:00 (23:00–23:59)	0.41960 ***	−0.17244 ***	0.59368 ***	0.19632 *	−0.12307 **	0.32278 **
Activity type (Ref. Working)						
Subsistence farming				0.02315	−0.01521	0.03742
Preparing food				0.17591 ***	−0.11141 ***	0.28553 ***
Doing housework				0.17709 ***	−0.11248 ***	0.28731 ***
Watching children				0.31481 ***	−0.05592 **	0.36788 ***
Shopping				0.31719 ***	−0.15436 ***	0.46938 ***
Commuting				0.26713 ***	−0.15690 ***	0.42178 ***
Rest (includes tea/coffee break)				0.60046 ***	−0.22492 ***	0.82634 ***
Chatting with someone				0.60203 ***	−0.21490 ***	0.81470 ***
Playing (includes cards/mahjong)				0.56535 ***	−0.17866 ***	0.74284 ***
Reading				0.46155 ***	−0.10445 ***	0.56678 ***
Watching TV				0.60435 ***	−0.22045 ***	0.82234 ***
Exercising or leisurely walk				0.62064 ***	−0.17605 ***	0.79542 ***
Other leisurely activity				0.65693 ***	−0.21444 ***	0.86672 ***
Grooming or bathing (self)				0.55377 ***	−0.20696 ***	0.75826 ***
Eating				0.43716 ***	−0.18821 ***	0.62492 ***
Other activity				0.06179	0.04014	0.02432
The quality of social interaction (Ref. Alone)						
Friendly				0.06322 ***	−0.00089	0.06286 ***
Irritating				−0.34967 ***	0.28857 ***	−0.63014 ***
Sample size	13,166	13,171	13,152	13,166	13,171	13,152

Note: *** *p* < 0.001, ** *p* < 0.01, * *p* < 0.05. ^@^ Ref. represented the reference group. ^#^ IWI: International Wealth Index.

## Data Availability

All data analyzed during this research can be acquired from J.S. and J.W.

## Appendix A

**Table A1:** Study areas and participants.

Province	County	Township	Village	Household	Participant
Shandong	Chiping	4	16	439	732
Shandong	Caoxian	4	16	456	770
An’hui	Linquan	4	16	454	704
Sichuan	An’yue	4	16	420	641
Total		16	64	1769	2847

**Table A2:** The distribution of three sets of abbreviated Day Reconstruction Method (DRM) questionnaire across counties.

County	Three Sets of Abbreviated DRM Questionnaire			Total
	Set A (Morning)	Set B (Afternoon)	Set C (Evening)	
Chiping	256	248	228	732
Caoxian	275	254	241	770
Linquan	242	231	231	704
Anyue	238	215	188	641
Total	1011	948	888	2847

**Table A3:** Results of multilevel analyses of time of day (ToD) and seven emotions.

Time of Day	Model 1						
(Ref. 4:00 (4:00–4:59)) ^@^	Worried	Rushed	Irritated/Angry	Depressed	Tense/Stressed	Calm/Relaxed	Enjoying
5:00 (5:00–5:59)	−0.06105	0.03733	0.01233	0.00337	0.02292	0.02367	0.02456
6:00 (6:00–6:59)	−0.09242 *	0.00279	−0.01392	−0.01853	−0.01889	0.01965	0.0198
7:00 (7:00–7:59)	−0.09494 *	−0.02271	−0.00374	−0.01314	−0.01646	0.0556	0.09828
8:00 (8:00–8:59)	−0.06593	0.04358	0.01377	0.01924	0.01392	0.02156	0.02884
9:00 (9:00–9:59)	−0.08313 *	0.05996	0.03167	0.00618	0.03218	−0.00835	0.02581
10:00 (10:00–10:59)	−0.08000 *	0.00109	−0.0126	−0.00207	−0.01928	0.03184	0.07269
11:00 (11:00–11:59)	−0.11311 **	−0.09963	−0.01761	−0.01509	−0.04014	0.11548	0.16448 *
12:00 (12:00–12:59)	−0.11260 **	−0.12607 *	−0.01397	−0.02105	−0.04305	0.14740 *	0.22383 **
13:00 (13:00–13:59)	−0.10580 **	−0.17303 **	−0.03154	−0.0459	−0.06034	0.15214 *	0.25920 **
14:00 (14:00–14:59)	−0.09949 **	−0.07532	0.00169	−0.0006	−0.03151	0.08742	0.1519
15:00 (15:00–15:59)	−0.07546	0.00238	0.03213	0.00465	0.02394	0.03623	0.09848
16:00 (16:00–16:59)	−0.0662	0.05811	0.0315	−0.00361	0.00507	−0.03317	−0.01229
17:00 (17:00–17:59)	−0.11650 **	0.0009	−0.01514	−0.04223	−0.03853	0.043	0.04909
18:00 (18:00–18:59)	−0.13321 **	−0.12940 *	−0.01004	−0.04218	−0.05931	0.08211	0.13965
19:00 (19:00–19:59)	−0.15423 ***	−0.19770 **	−0.04298	−0.06644	−0.08169 *	0.22454 **	0.31324 ***
20:00 (20:00–20:59)	−0.16715 ***	−0.25350 ***	−0.05616	−0.08580 *	−0.09593 *	0.25492 ***	0.35013 ***
21:00 (21:00–21:59)	−0.15898 ***	−0.26338 ***	−0.06537	−0.09118 *	−0.08787 *	0.31083 ***	0.43671 ***
22:00 (22:00–22:59)	−0.16014 ***	−0.25793 ***	−0.05796	−0.10356 *	−0.10017 *	0.31045 ***	0.47219 ***
23:00 (23:00–23:59)	−0.12408 *	−0.30945 ***	−0.07622	−0.14083 **	−0.12446 *	0.34870 ***	0.46076 ***
**Time of day**	**Model 2**						
(**Ref. 4:00 (4:00–4:59)) ^@^**	**Worried**	**Rushed**	**Irritated/angry**	**Depressed**	**Tense/stressed**	**Calm/relaxed**	**Enjoying**
5:00 (5:00–5:59)	−0.0576	0.01819	0.01028	0.00466	0.02276	0.03853	0.04852
6:00 (6:00–6:59)	−0.09874 **	−0.03611	−0.02488	−0.02284	−0.0255	0.0582	0.06677
7:00 (7:00–7:59)	−0.10491 **	−0.04271	−0.01518	−0.01827	−0.02171	0.07669	0.11186
8:00 (8:00–8:59)	−0.09045 *	−0.01707	−0.01178	0.0029	−0.00389	0.06329	0.07082
9:00 (9:00–9:59)	−0.10603 **	−0.01277	0.0067	−0.00487	0.0145	0.0346	0.0743
10:00 (10:00–10:59)	−0.09074 *	−0.02382	−0.02458	−0.00314	−0.02546	0.0397	0.07211
11:00 (11:00–11:59)	−0.10794 **	−0.06219	−0.01283	−0.00422	−0.03139	0.08535	0.11028
12:00 (12:00–12:59)	−0.10174 **	−0.05279	−0.0008	−0.00584	−0.02669	0.09186	0.12806
13:00 (13:00–13:59)	−0.09594 *	−0.08409	−0.01644	−0.02642	−0.04119	0.06617	0.12625
14:00 (14:00–14:59)	−0.10491 **	−0.04627	0.00146	0.00902	−0.02687	0.03829	0.08045
15:00 (15:00–15:59)	−0.09900 *	−0.04324	0.01347	−0.00178	0.00955	0.04347	0.10527
16:00 (16:00–16:59)	−0.09897 *	−0.06045	−0.00527	−0.02416	−0.02411	0.0399	0.08315
17:00 (17:00–17:59)	−0.13817 ***	−0.09517	−0.04206	−0.05661	−0.05999	0.10316	0.13012
18:00 (18:00–18:59)	−0.13109 **	−0.12904 *	−0.01202	−0.03459	−0.05639	0.07243	0.12281
19:00 (19:00–19:59)	−0.13956 ***	−0.11889 *	−0.02479	−0.04381	−0.06112	0.14130 *	0.18967 **
20:00 (20:00–20:59)	−0.14709 ***	−0.14832 **	−0.02951	−0.05354	−0.06745	0.12164	0.16710 *
21:00 (21:00–21:59)	−0.13497 **	−0.14744 *	−0.02861	−0.04759	−0.05331	0.13152	0.20855 **
22:00 (22:00–22:59)	−0.13387 **	−0.13267 *	−0.01586	−0.0556	−0.06343	0.1008	0.21317 **
23:00 (23:00–23:59)	−0.10948 *	−0.19289 *	−0.03993	−0.09988	−0.09532	0.15273	0.21999 *
Sample size	13,186	13,184	13,183	13,184	13,177	13,180	13,169

Note: (1) *** *p* < 0.001, ** *p* < 0.01, * *p* < 0.05. ^@^ Ref. represented the reference group. (2) For model 1, we controlled for county, sets of DRM questionnaire, and sociodemographic factors, including gender, age, age^2^, highest education level completed, marital status, occupation, self-perceived health and International Wealth Index (IWI); for model 2, we further controlled for activity type and the quality of social interaction on the basis of model 1.
